# The gnathosoma is a bad character rather than evidence for mite monophyly

**DOI:** 10.1098/rspb.2025.0368

**Published:** 2025-04-30

**Authors:** Samuel J. Bolton

**Affiliations:** ^1^ Florida State Collection of Arthropods, Division of Plant Industry, Florida Department of Agriculture and Consumer Services, Gainesville, FL 32608, USA

**Keywords:** mite, Acari, Arachnida, Acariformes, Parasitiformes, gnathosoma

## Abstract

In recent years, the case for the monophyly of mites or Acari (Parasitiformes + Acariformes) has looked increasingly weak. Much of the remaining doubt about the artificiality of this taxon stems from the importance long attributed to the gnathosoma, widely considered the most convincing morphological character supporting monophyly. The gnathosoma has long been interpreted as originating via the fusion together of the palpal coxae, which is thought to have contributed to the consolidation of the mouthparts into a compact feeding apparatus that articulates as a single unit. However, an investigation of the mouthparts of Acariformes, reported herein, revealed that fusion together of the palpal coxae is an uncommon state that convergently evolved in multiple acariform taxa rather than evolving only once, as a synapomorphy uniting Acariformes and Parasitiformes. Moreover, other defining features of the gnathosoma involve either very different modifications or structures that are not homologous between both main lineages of mites. Therefore, the gnathosoma is a bad character—poorly defined and based on a series of misinterpretations—that should not be treated as evidence for mite monophyly.

## 1. Introduction

Mites (Acariformes + Parasitiformes), which include ticks (Ixodida), represent a megadiverse group of arachnids that has, more often than not, been treated as a single taxon: Acari [1–5]. However, mites are probably not a natural group. The majority of phylogenomic analyses have recovered mites as non-monophyletic [6–11], and the few occasions when mite monophyly has instead been recovered [10,12,13] can be readily attributed to taxonomic undersampling (overtly problematic for two studies [10,13]) and long-branch attraction [8,11,14]. A high proportion of the most taxonomically comprehensive phylogenomic analyses have recovered Acariformes as sister to a clade comprising the rest of Arachnida and Xiphosura [8,11].

**Figure 1.**
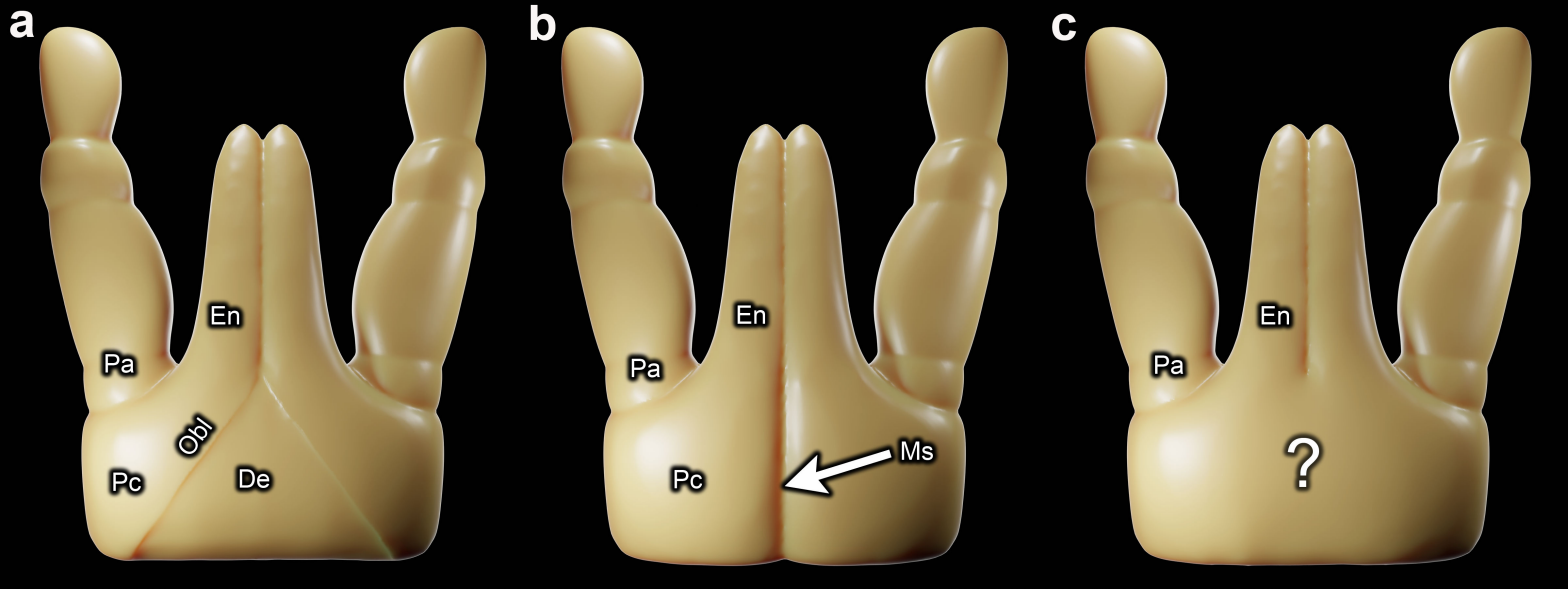
Schematic three-dimensional representations of the palps and subcapitulum in mites: (*a*) palpal coxae separated by a triangular-shaped deutosternum (sternite of somite II), in accordance with Weigmann’s [15] interpretation of the stenarthric form of subcapitulum in Oribatida; (*b*) palpal coxae fused together; (*c*) state unknown because the inner margins of the palpal coxae are not visible (the condition of most mites). De = deutosternum; En = endite; Ms = medial suture; Obl = oblique suture delineating border between palpal coxa and deutosternum; Pa = palp (excluding coxa and endite); Pc = palpal coxa.

Much of the remaining doubt about the artificiality of mites stems from the importance long attributed to the gnathosoma (see glossary in electronic supplementary material, table S1), a compact feeding apparatus that articulates against the main body. The gnathosoma includes the chelicerae, which are dorsal appendages for grabbing or piercing. The ventral-most region of the gnathosoma is formed from the palps and subcapitulum (palpal coxae, endites and part of somite II) (figure 1). In their review on the relationship between Acariformes and Parasitiformes, Dunlop & Alberti mention that the gnathosoma is widely thought to be the most convincing character in support of mite monophyly [16]. The gnathosoma has also been used to support the monophyly of Acaromorpha (Acari + Ricinulei) [17], but there is considerable scepticism about the presence of a true gnathosoma in Ricinulei because the mouthpart region of this taxon is weakly integrated and relatively immobile [18].

The gnathosoma remains pertinent to the ongoing controversy on the monophyly of Acari [19]. In the discussion of a recently published phylogenomic analysis that recovered this relationship, the gnathosoma was the only mentioned morphological character that was considered to represent a genuine autapomorphy for Acari [12]. Other morphological characters that have been used to support mite monophyly have been found to be either problematic in their interpretation or unpersuasive owing to homoplasy [20].

The gnathosoma appears to provide a strong argument for mite monophyly because it is a complex feeding apparatus that is difficult to reconcile with homoplasy. It has long been thought that the formation of the gnathosoma proceeded through the expansion and fusion together of the palpal coxae at the midline [1,3,16,18,21–30]. It is generally believed that as a result of this process, somite II and its sternite—the deutosternum—have been obliterated in all mites (figure 1a,b). This is clearly implied when the gnathosoma is described as being formed from only appendages [26] or only somite I, the chelicerae and the biramous appendages of somite II [29].

However, determining if the palpal coxae are fused together is complicated by the lack of any visible trace of the inner margins of the palpal coxae in most mite taxa (figure 1c). Although the absence of visible borders is consistent with fusion, this does not specifically indicate that the palpal coxae are fused together. The palpal coxae may instead be fused to somite II (figure 1a). Fusion of the palpal coxae together (‘together’ is henceforth omitted for brevity) is evident from a medial suture that runs along the length of the ventral face of the subcapitulum (figure 1b). This suture, which is exhibited by only a small proportion of mite taxa, probably forms the border between the expanded palpal coxae.

Based on a medial suture, fusion of the palpal coxae is clearly evident in Parasitiformes, specifically Holothyrida [31,32] and at least some Uropodina [33]. In Acariformes, perhaps Cheyletidae [34] and Erythraeidae [35] provide the most convincing evidence for fused palpal coxae, which is revealed by a medial suture or apodeme (electronic supplementary material, figure S2a,b). However, Weigmann hypothesized that the stenarthric subcapitulum in Oribatida is consistent with the presence of a deutosternum between the palpal coxae [15]. According to this interpretation, a pair of oblique sutures (Obl) delineate the border between the deutosternum and the palpal coxae (figure 1a). Weigmann further hypothesized that the presence of a deutosternum may be plesiomorphic to all mites. This appears incongruent with the subcapitulum of the acariform mite *Terpnacarus gibbosus* (Womersley), which has a medial suture and thus fused palpal coxae [36]. This species is potentially important for reconstructing the ancestral state of the subcapitulum because it falls within Endeostigmata, a grade that comprises the majority of the most deeply rooted acariform taxa [37,38]. But the examination of the mouthparts of other Endeostigmata, reported herein, revealed that fusion of the palpal coxae is an uncommon state that has convergently evolved in multiple acariform taxa rather than evolving only once, as a synapomorphy uniting Acariformes and Parasitiformes.

## 2. Results and discussion

### (a) Subcapitulum

**Figure 2.**
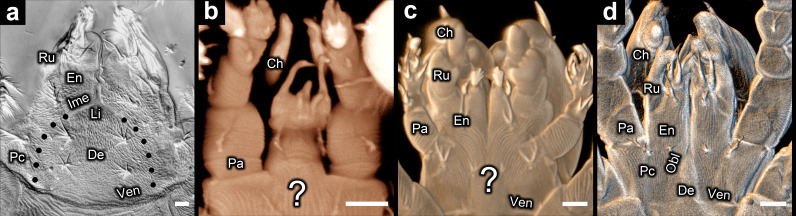
Subcapitulum in Endeostigmata (differential interference contrast (DIC) microscopy and confocal laser scanning microscopy (CLSM)): (a) *Alycus* sp. (DIC) (dotted lines represent the hypothesized border of the deutosternum); (b) *Nanorchestes* sp. 1 (CLSM); (c) *Micropsammus* sp. 1 (CLSM); (d) *Stigmalychus* sp. (CLSM). Large question mark = unknown if palpal coxae fused or separate; Ch = chelicera; De = deutosternum (evident in *Alycus* from oblique inner margins of endites); En = endite; Ime = inner margin of endite (oblique part); Li = deutosternal labium; Obl = oblique suture delineating border between palpal coxa and deutosternum; Pa = palp (excluding coxa and endite); Pc = palpal coxa; Ru = rutellum; Ven = ventrocapitular furrow; scale bars = 5 µm.

**Figure 3.**
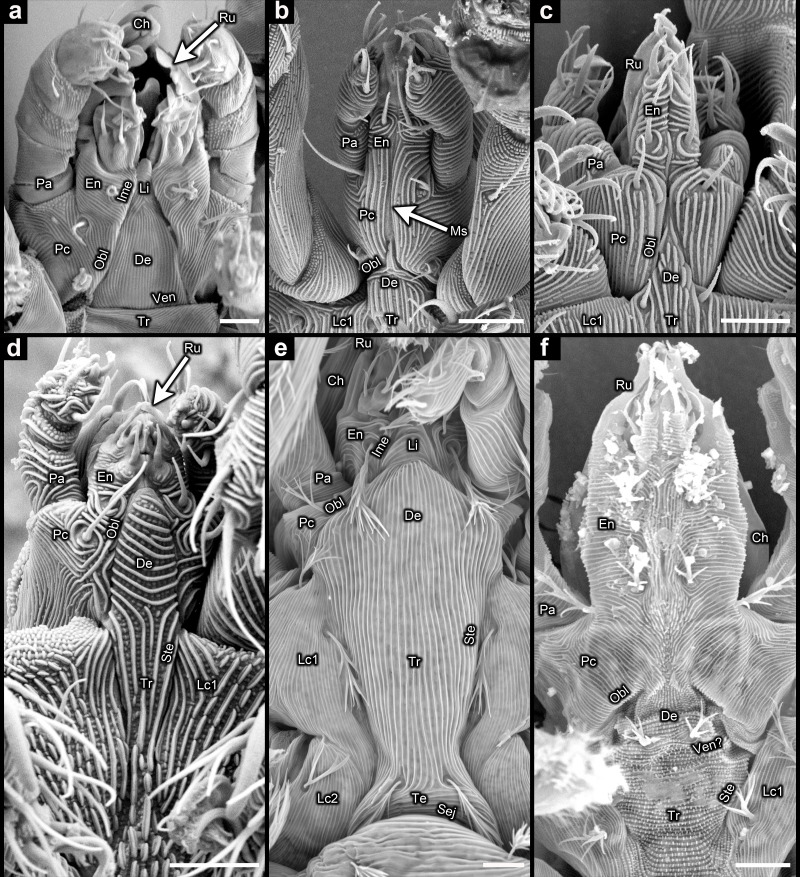
Subcapitulum in Endeostigmata (scanning electron microscopy): (a) *Proteonematalycus wagneri* Kethley; (b) *Cunliffea strenzkei* (Cunliffe); (c) *Gordialycus* sp.; (d) *Osperalycus tenerphagus* Bolton and Klompen; (e) *Caenonychus* sp.; (f) *Oehserchestes* sp. Ch = chelicera; De = deutosternum; En = endite; Ime = inner margin of endite; Lc1 = leg coxa I; Lc2 = leg coxa II; Li = deutosternal labium; Ms = medial suture between fused palpal coxae; Obl = oblique suture delineating border between palpal coxa and deutosternum; Pa = palp (excluding coxa and endite); Pc = palpal coxa; Ru = rutellum; Sej = sejugal furrow; Ste = sternal border; Te = tetrasternum; Tr = tritosternum; Ven = ventrocapitular furrow; scale bars = 5 µm.

The inner margins of the palpal coxae are indiscernible—neither a medial suture nor a pair of oblique sutures is evident—in some Endeostigmata, namely *Alycus*, *Nanorchestes* and *Micropsammus* (figure 2a–c). The ventrocapitular furrow (Ven) (see glossary in electronic supplementary material, table S1) is absent in Nematalycidae (*Cunliffea*, *Gordialycus*, *Osperalycus*) (figure 3b–d) and *Caenonychus* (figure 3e), whereas it is very faint, if present at all, in *Oehserchestes* (figure 3f). Note that the ventrocapitular furrow of *Nanorchestes* (figure 2b) is out-of-frame rather than absent (electronic supplementary material, figure S3a,b) [39,40].

The oblique sutures (Obl) that define the stenarthric form in Oribatida (figure 1a) are also present in other taxa within Acariformes (figures 2d and 3). The sternum of *Osperalycus*, *Caenonychus* and *Oehserchestes* is clearly delimited by a sternal border (Ste in figure 3d–f), which runs from the inner margins of the leg coxae to the inner margins of the palpal coxae, forming the oblique sutures that define the stenarthric state. Therefore, the oblique sutures of these and other acariform taxa, including those found in Oribatida [15], correspond to the anterior border of the deutosternum. Furthermore, in *Osperalycus* and *Caenonychus* the presence of a deutosternum is unambiguous because the sternum, which includes the deutosternum, is not divided up transversely by a ventrocapitular furrow (figure 3d,e). If a ventrocapitular furrow were present in all acariform mites, a case could possibly be made that this furrow delineates the anterior border of the sternum, and the oblique sutures could instead be explained as novel features that are unrelated to segmentation. Although a very faint ventrocapitular furrow may be present in *Oehserchestes*, the presence of a deutosternum is no less obvious in this taxon because the integument of the deutosternum (De) shares the same pattern of tuberculated striae (ridges) as the rest of the sternum, including the tritosternum (sternite of somite III) (Tr), whereas the coxae of the legs and palps (Pc and Lc1) are smoothly striated (figure 3f). For these reasons, these three taxa remove any remaining doubt about the presence of a deutosternum in some Acariformes.

**Figure 4.**
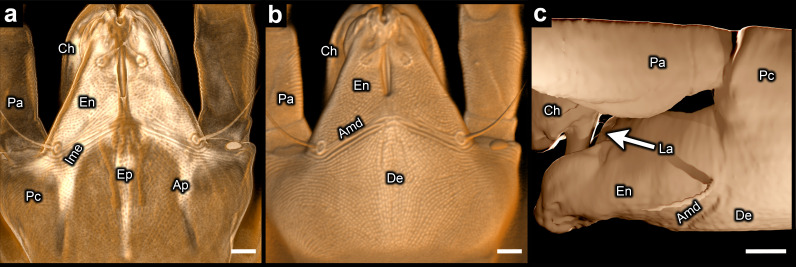
Infracapitulum of *Tydeus* sp. (confocal laser scanning microscopy) (all images portray different representations of the same specimen): (a) ventral view of translucent representation (showing internal morphology of infracapitulum); (b) ventral view of opaque representation (showing ventral face of subcapitulum); (c) lateral view of mesh model (showing external morphology of infracapitulum). Amd = anterior margin of deutosternum; Ap = palp coxal apodeme; Ch = chelicera; De = deutosternum; En = endite; Ep = strongly fluorescent region associated with epistome (dorsal part of the infracapitulum); Ime = inner margin of endite (oblique part); La = labrum; Pa = palp (excluding coxa and endite); Pc = palpal coxa; scale bars = 5 µm.

With the single exception of *Cunliffea*, fused palpal coxae were not evident in any of the Endeostigmata that were observed. In *Cunliffea*, the deutosternum is extremely short, accommodating the fusion of the palpal coxae along the midline. Hence, there is a medial suture (Ms) along a large proportion of the ventral face of the subcapitulum (figure 3b). The deutosternum is more prominent in the other taxa (figures 2d, 3a,c–f and 4b), so that the palpal coxae are separated along most or all of their length. There is a meeting point between the palpal coxae in front of the deutosternum in *Gordialycus* (figure 3c) and possibly *Oehserchestes* (figure 3f), but this meeting point is too narrow for the palpal coxae to be considered fused.

In *Proteonematalycus* and *Caenonychus*, the deutosternum is sufficiently long for the anterior part to be wedged between the endites (figure 3a,e), where it forms a labium (lower lip). Homology between the labium and the anterior part of the deutosternum is obvious in these taxa because the edges of the labium represent continuations of the oblique sutures that delineate the borders between the deutosternum and the palpal coxae. A labium is also present in *Alycus* [41], although the inner margins of the palpal coxae are not visible in this genus. Instead, the inner margins of the endites (Ime) appear to end abruptly where they meet the base of the labium (figure 2a). Nonetheless, they must continue on, posteriorly, where they extrapolate as borders between the palpal coxae and a prominent deutosternum (dotted lines in figure 2a). Moreover, no mite is known to exhibit fused palpal coxae in combination with a labium. Therefore, the presence of a labium strongly suggests that a deutosternum separates the palpal coxae.

Of all the taxa examined, *Tydeus* represents the most divergent morphology. When the confocal three-dimensional model of this genus is made translucent (figure 4a), the separation of the palpal coxae is evident from the convergence of the inner margins of the strongly fluorescent endites (Ime). When the palpal coxae are instead fused, the inner margins of the endites run completely parallel along the midline rather than converge (figure 1b). Separation of the palpal coxae is also evident in *Tydeus* from the palp coxal apodemes (Ap in figure 4a). Apodemes are known to delineate the margins of segments in other arthropods [42], including the inner margins of leg coxae in various mite groups, e.g. Astigmata and Heterostigmata. The palp coxal apodemes of *Tydeus* are therefore very likely to delineate the inner margins of the palpal coxae, thus indicating the palpal coxae are separate.

Although the borders between the palpal coxae and the deutosternum are indiscernible in *Tydeus*, a suture (Amd) that traverses the ventral face of the subcapitulum is clearly visible in an opaque representation of the three-dimensional model (figure 4b). This suture almost certainly represents the anterior margin of the deutosternum. The palps have shifted to a relatively dorsal position (figure 4c), and so part of the deutosternum is beneath the basal portions of the endites, which project obliquely down from the palpal coxae. Consequently, the bases of the endites are not visible in the ventral view of the opaque rendering, being concealed from below by the deutosternum (figure 4b). The only visible sections of the endites are the parts that project out beyond the anterior margin of the deutosternum. The same basic morphology may be present in the diarthric subcapitulum in Oribatida, where the palps project out from a distinctly dorsal position (fig. 13 in Lindo *et al*. [43]; fig. 15 in Fernandez *et al*. [44]). In oribatids, diarthric sutures traverse the endites at the same approximate position as the Amd suture in *Tydeus*. These similarities appear to represent a suite of convergences that are associated with dorsoventral deepening of the infracapitulum (epistome, labrum and subcapitulum).

**Figure 5.**
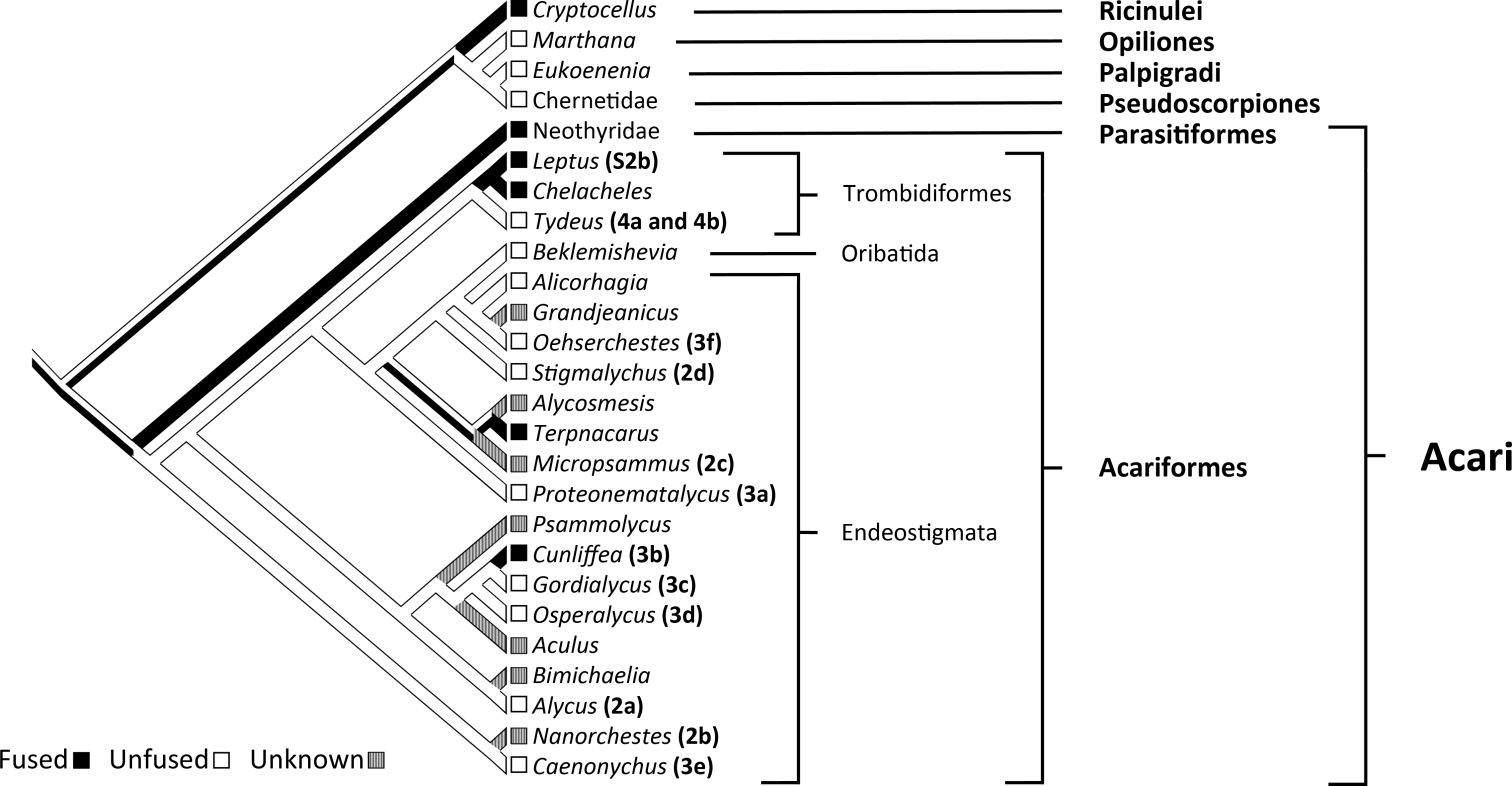
Phylogenetic trace (parsimony-based reconstruction) for the state of the palpal coxae in a tree constrained for mite monophyly: a simplified version of a maximum likelihood tree (rRNA: 18S and 28S; amino acids: COI, HSP70 and SRP54) (electronic supplementary material, treefile S4), based on identical methods and data to a recent phylogenetic analysis [38], but with Acari constrained to be monophyletic. Numbers and letters in parentheses, following the names of some genera, refer to figures herein. The unfused state of *Alicorhagia* and *Alycus* is based on the presence of a deutosternal labium [41,45]. State assignment in all other mites is based on direct observations (differential interference contrast microscopy) and/or the taxonomic literature [32,34,36,46–50]. Endeostigmata are defined in accordance with Beaulieu *et al*. [51].

Clearly, not all mites have fused palpal coxae. Moreover, fusion of the palpal coxae is uncommon in Acariformes. Even when topology is constrained for mite monophyly and Parasitiformes are coded as having only fused palpal coxae (but see the next paragraph), fusion in Acariformes is recovered as convergent rather than as symplesiomorphic with Parasitiformes (figure 5). Accordingly, the monophyly of mites is not supported by fused palpal coxae.

**Figure 6.**
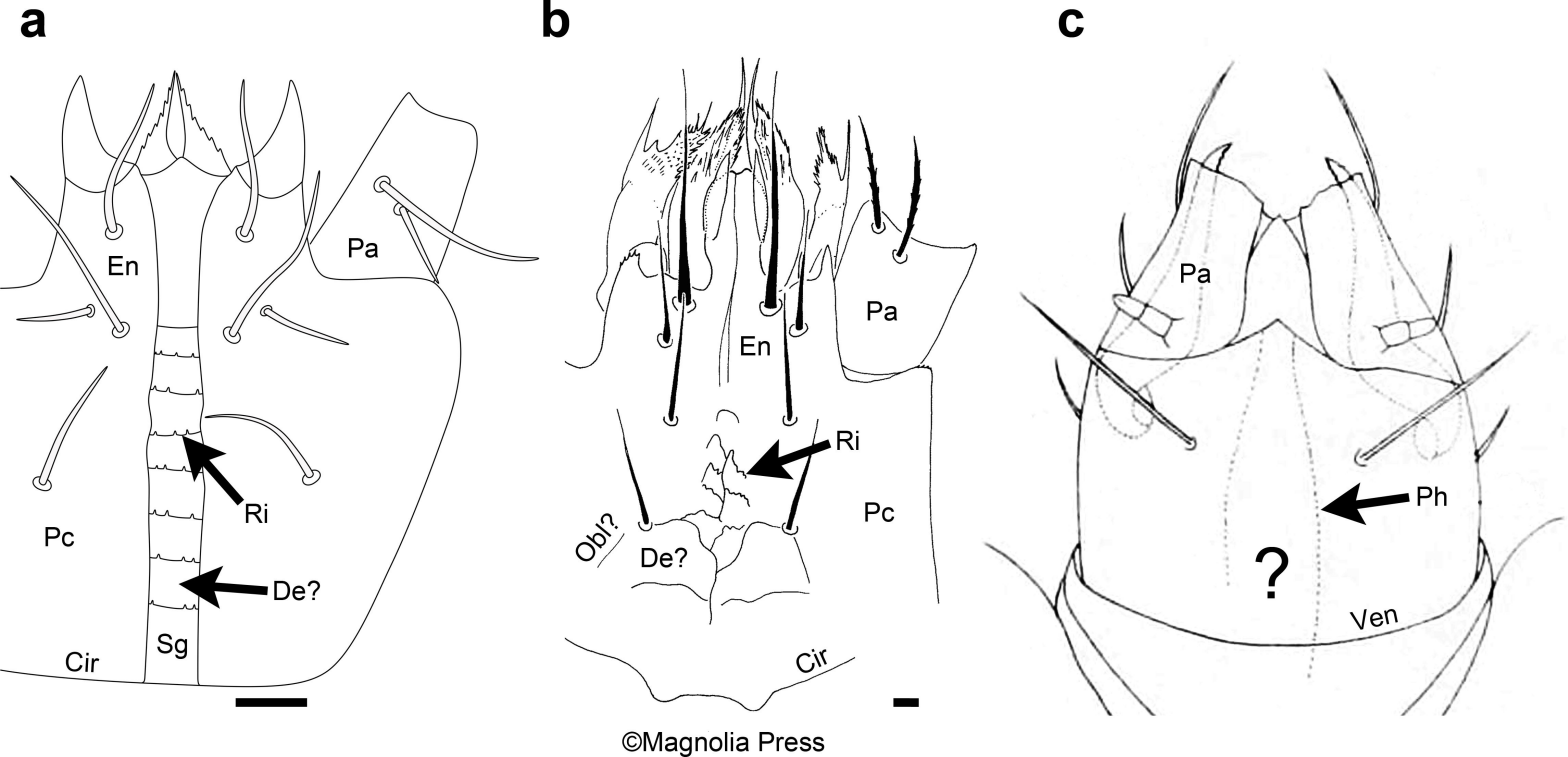
Drawings of the subcapitulum: (a) *Asca* sp. (b) *Messoracarus schwendingeri* Kontschán & Seeman (copied from Kontschán & Seeman [54]; reproduced with permission from the copyright holder, Magnolia Press; labels added); (c) *Siteroptes graminum* (Reuter) (copied from Reuter [24]; labels replaced with new ones). Large question mark = unknown if palpal coxae fused or separate; Cir = circumcapitular furrow; De = deutosternum; En = endite; Obl = oblique suture delineating border between palpal coxa and deutosternum; Pa = palp (excluding coxa and endite); Pc = palpal coxa; Ph = pharynx outline; Ri = ridge that is jagged or toothed; Sg = subcapitular groove; Ven = ventrocapitular furrow; scale bars = 5 µm (not available for *Siteroptes graminum*).

It is likely that some or even many species of Parasitiformes also have separate palpal coxae but only ventrally (see discussion of the gnathotectum below). The subcapitular groove of Gamasina has been homologized with a narrow deutosternum [52,53], separating the palpal coxae completely along the subcapitulum (figure 6a). However, this hypothesis has obviously not gained wide acceptance. This is perhaps because the subcapitular groove has a clear function [55], which may mean it is a novel and adaptive structure that bears no relationship to segmentation. But homology between this groove and a deutosternum should be reconsidered based on the subcapitulum of *Messoracarus schwendingeri* Kontschán & Seeman (figure 6b). The subcapitulum of this species is depicted, in the description, as bearing oblique sutures [54], giving the appearance of the triangular deutosternum that is present in Acariformes (figure 1a). The fine ridges between the oblique sutures are jagged in places, thus resembling the toothed ridges along the subcapitular groove in Gamasina (figure 6a).

### (b) History of the treatment of the subcapitulum

In the early nineteenth century, Savigny proposed that the insect labium (not homologous with the labium of mites) is formed through the fusion of a pair of appendages [56]. This has since been confirmed through studies of homeotic genes [57–59]. Late nineteenth to early twentieth century entomologists, chiefly acarologists, hypothesized that an analogous modification arose in mites (thought by some authors to be closely related to insects [60,61]) through the fusion of the palps [21–23,62–64], which were mistakenly homologized with the maxillae of insects [60,61,63,64]. In Arachnida, the somite that bears the palps is instead homologous with the limbless intercalary segment of insects [65].

Nonetheless, early entomologists were largely correct to infer that mites have a functional lower lip that is formed from the palps, specifically the endites [66]. Only a few mite species have a deutosternal labium between the endites. In the vast majority of mite species, either the endites are fused together or there is a slit between them so that they appear close to a state of fusion. Therefore, in taxa in which the inner margins of the palpal coxae are not visible (figure 1c), which is the case in most mite species, it was intuitive for early acarologists, e.g. Brucker [21] for the diarthric subcapitulum in Oribatida, to assume that the medial border or slit between the endites was the continuation of a medial border between fused palpal coxae (figure 1b). However, the border/slit between the endites can instead begin at or in front of the anterior margin of a deutosternum that separates the palpal coxae (figures 1a and 4b). But this possibility does not appear to have been considered. Without an alternative way of conceiving the subcapitulum, the absence of visible borders was sometimes treated as exhibiting the state of fused palpal coxae. This is how Reuter interpreted the subcapitulum of *Siteroptes graminum* (Reuter) [24]. The inner margins of the palpal coxae of this species are indiscernible, as depicted by Reuter himself (figure 6c). Rather than viewing this as ambiguous, he asserted that it is easy to recognize that the palpal coxae are fused together at the midline [24].

Based on the interpretation of only a small selection of species, fusion of the palpal coxae was assumed for all mites in the first decade of the twentieth century [21–24]. This has remained the dominant hypothesis on the makeup of the subcapitulum, although a number of acarologists have since inferred the presence of a deutosternum between the palpal coxae in many, if not all, mite taxa [2,15,52,53,67,68]. But in the most widely cited textbooks on mites, it is commonly reasserted that all mites have fused palpal coxae [1,3,26,29]. Fusion is also assumed for all mites in morphology-based phylogenetic analyses undertaken for Arachnida [18,27,30,69]. This may be largely because the presence of a deutosternum has not seemed sufficiently obvious in any mite. As discussed above, any remaining ambiguity about the presence of a deutosternum is ended by *Osperalycus*, *Oehserchestes* and *Caenonychus* (figure 3d–f).

### (c) A bad character

**Figure 7.**
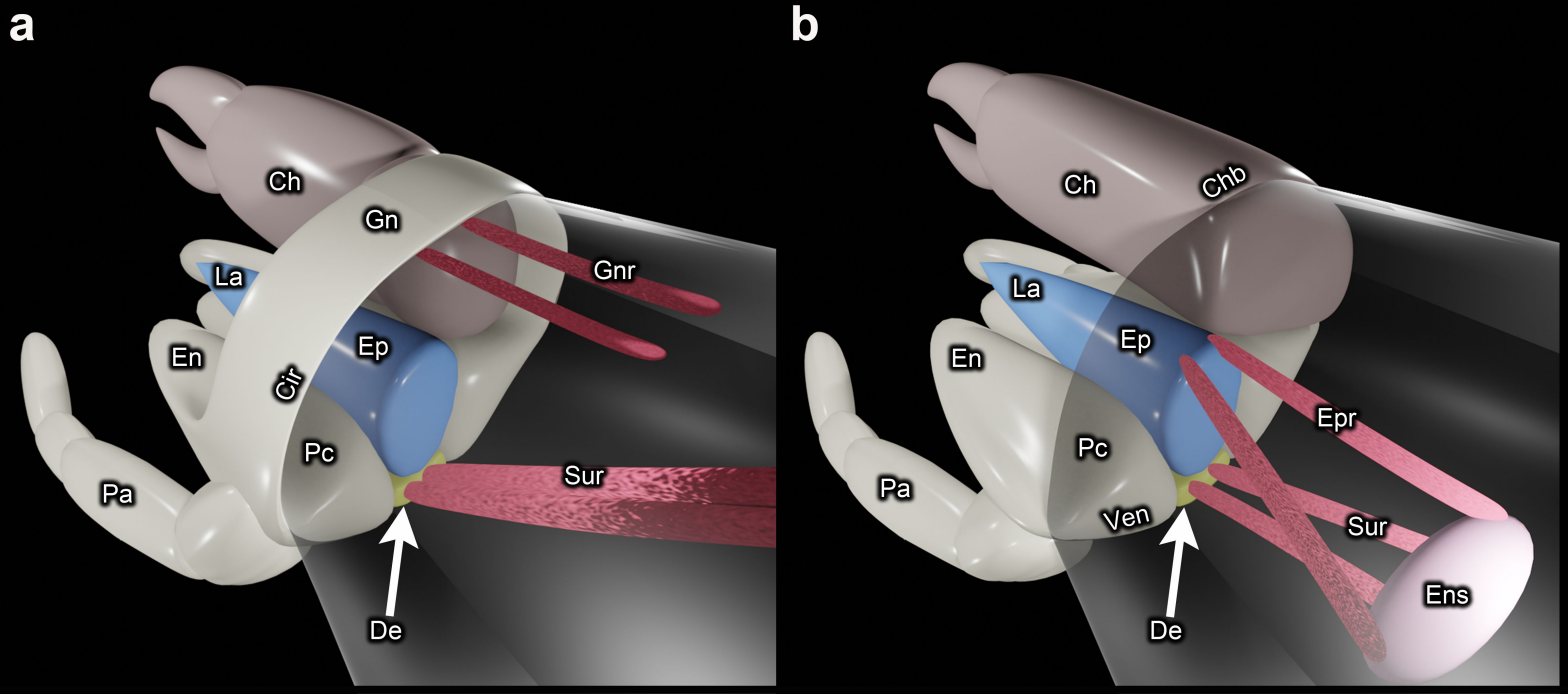
Schematic three-dimensional representations illustrating the upward and downward articulation of the mouthpart region: (a) Parasitiformes; (b) Acariformes. The wall of the main body is transparent. Ch = chelicera (left one is omitted to improve the visibility of the important structures); Chb = border between chelicera and main body; Cir = circumcapitular furrow; De = deutosternum; En = endite; Ens = endosternum; Ep = epistome; Epr = epistomal retractor muscle; Gn = gnathotectum; Gnr = gnathotectal retractor muscle; La = labrum; Pa = palp (excluding coxa and endite); Pc = palpal coxa; Sur = subcapitular retractor muscle (may not be homologous between Acariformes and Parasitiformes); Ven = ventrocapitular furrow.

**Figure 8.**
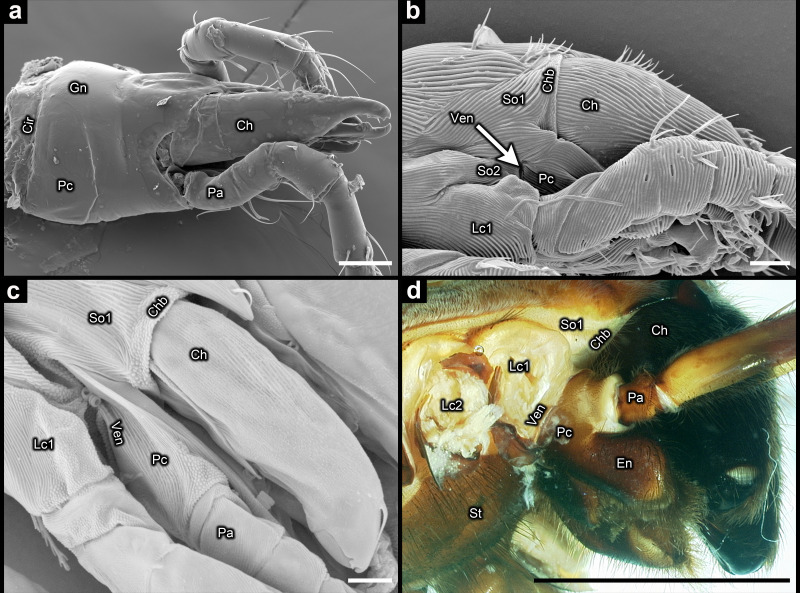
Circumcapitular and ventrocapitular furrows: (a) *Macrocheles muscaedomesticae* (Scopoli) (Parasitiformes) (scanning electron microscopy (SEM)) (mouthparts removed from the body to make the circumcapitular furrow visible); (b) *Micropsammus* sp. 2 (Acariformes) (SEM); (c) *Proteonematalycus wagneri* Kethley (Acariformes) (SEM); (d) *Hogna lenta* (Hentz) (Araneae) (dissection microscope). Ch = chelicera; Chb = border between chelicera and somite I; Cir = circumcapitular furrow; En = endite; Gn = gnathotectum; Lc1 = leg coxa I; Lc2 = leg coxa II; Pa = palp (excluding coxa and endite); Pc = palpal coxa; So1 = somite I; So2 = somite II; St = sternum; Ven = ventrocapitular furrow; scale bars = 50 µm for (a); 5 µm for (b,c); 5 mm for (d).

In their important review on the monophyly of Acari, Dunlop & Alberti [16, page 2] stated that the gnathosoma is ‘widely regarded as the best character supporting a monophyletic Acari’. The gnathosoma has long been interpreted as originating via the fusion of the palpal coxae [24,29]. For this reason, the fusion of the palpal coxae is treated as one of the defining attributes of the gnathosoma [18,30]. Defined using only this attribute, the gnathosoma does not support mite monophyly (figure 5). However, the gnathosoma is also defined through its articulation as a single, integrated unit against the main body [18,28]. But based on that definition the gnathosoma again fails to support the monophyly of Acari because there are fundamental differences between Acariformes and Parasitiformes in the modifications that led to integration between the infracapitulum and the chelicerae, so that the mode of articulating the mouthpart region is also very different (figure 7a,b). In Parasitiformes, the mouthparts articulate as a single unit owing to the evolution of a gnathotectum, a structure that originated via the dorsomedial expansion of the outer walls of the palpal coxae up and around the chelicerae, fusing at the midline (Gn in figures 7a and 8a) [3].

Acariformes lack a gnathotectum. In a number of acariform mites (e.g. Endeostigmata other than Eriophyoidea and Nematalycidae) there is no integration between the chelicerae and infracapitulum, so that the latter can articulate without forcing the former to articulate with it. In other acariform mites the mouthparts articulate as a single unit but owing to very different modifications to a gnathotectum. In some acariform taxa, the chelicerae are integrated with the infracapitulum via subcapitular modifications but without dorsomedial fusion of the palpal coxae [70–72]. And in many species of Oribatida the mouthparts articulate as a single unit because the chelicerae and infracapitulum are tightly enclosed within a cavity, the camerostome, much like a ball and socket joint [73].

Therefore, when the mouthparts articulate as a single unit in Acariformes it is because of entirely different modifications to those of Parasitiformes. As already pointed out by van der Hammen [74], there are important differences in associated musculature, which he viewed as evidence that the gnathosoma evolved independently in Parasitiformes and Acariformes. The muscles that rotate the infracapitulum upwards in Parasitiformes are attached to the gnathotectum (Gnr in figure 7a) [20,74]. Upward rotation of the infracapitulum in Acariformes is instead via muscles attached to the dorsobasal edge of the epistome (Epr in figure 7b) [20,73]. Similarly oriented muscles are also attached to the dorsobasal edge of the epistome in Ricinulei, Opiliones and Scorpiones [75,76]. Moreover, in both Opiliones and Acariformes these muscles are posteriorly connected to a ventrally positioned endosternum [3,73,75]. This possibly indicates that the musculature associated with the articulation of the infracapitulum in Acariformes is symplesiomorphic with non-mite arachnids. And so, many species within Endeostigmata appear to have retained a plesiomorphic mode of articulating their mouthparts, with neither integration between the infracapitulum and chelicerae nor associated new musculature.

The gnathosoma is also sometimes defined by the presence of a circumcapitular furrow [77]. This is a furrow that encircles the base of the mouthpart region dorsoventrally, enabling the mouthparts to articulate as a single unit against the main body. But the dorsal part of this furrow is not homologous across mites. In Parasitiformes it is formed from the border between the gnathotectum and the main body (Cir in figures 7a and 8a), whereas in Acariformes there is no gnathotectum, and so the dorsal part of what is considered to be the circumcapitular furrow is formed from the borders between the chelicerae and the main body (Chb in figures 7b and 8b,c). These are plesiomorphic borders shared with all non-mite arachnid lineages (figure 8d).

The term ‘circumcapitular furrow’ may seem appropriate for many members of Acariformes because the posterior borders of the chelicerae (Chb) are often closely aligned with the posterior border of the subcapitulum (Ven). This can give the appearance of a single furrow (figure 7b). But aside from the aforementioned problem of homology, in many species of Endeostigmata this alignment is not very close (figure 8b,c), and it can be closer in non-mite arachnids, such as Araneae (figure 8d). Therefore, the term ‘circumcapitular furrow’ is not correct for Acariformes, and the term ‘ventrocapitular furrow’ (Ven) is herein used instead for this lineage, specifically for the ventrolateral border between the subcapitulum and the main body (figures 2a,c,d, 3a,f and 8b,c). A ventrocapitular furrow is also present in non-mite arachnids, including Araneae (figure 8d), because it is a plesiomorphic margin that runs ventrally along the border between somites II and III (including associated coxae), and laterally along the borders between the palpal coxae and somite II (figure 8b). The absence of a ventrocapitular furrow in some Endeostigmata—namely Nematalycidae, *Caenonychus* and possibly *Oehserchestes*—is rare and probably apomorphic.

Therefore, there is no available definition of the gnathosoma that can allow this structure to provide support for the monophyly of Acari. If defined only by the condition of having fused palpal coxae, the gnathosoma would have convergently evolved in multiple acariform taxa rather than evolving only once as a synapomorphy uniting Acariformes and Parasitiformes (figure 5). However, Thelyphonida and Schizomida are considered to lack a gnathosoma although they have fused palpal coxae [18,30]. This is because the gnathosoma is also defined by its articulation as a single unit against the main body [18]. But the modifications that brought about this articulation are completely different between Acariformes and Parasitiformes. Moreover, all Acariformes lack a true circumcapitular furrow (figure 8b,c). And excluding Eriophyoidea (*Aculus* in figure 5) and Nematalycidae (*Cunliffea*, *Goridalycus*, *Osperalycus* and *Psammolycus* in figure 5), Endeostigmata also lack any integration of the infracapitulum with the chelicerae, so that their mouthparts articulate no differently from non-mite arachnids. The term ‘gnathosoma’ is therefore misleading when applied to all mites, and it seems unlikely that the gnathosoma can be salvaged via a new definition, such as the presence of a gnathotectum, which is only present in Parasitiformes. One of the principal reasons for using the term 'gnathosoma' is to underscore that Acari represent a distinct lineage from all other arachnids [4,24]. Accordingly, arguments for mite monophyly and an autapomorphic gnathosoma have sustained each other, in a circular way, for more than a century. For all these reasons, the gnathosoma is a bad character—poorly defined and based on a series of misinterpretations—that should not be treated as evidence for mite monophyly.

## 3. Methods

Images of acariform mites were generated over the course of more than a decade (2013−2024) using scanning electron microscopy (SEM), confocal laser scanning microscopy (CLSM), differential interference contrast (DIC) microscopy and dissection microscopy. Specimen information is provided in electronic supplementary material, table S5. The following microscopes were used: (i) Hitachi S-4700 field emission cryo-SEM: *Cunliffea strenzkei* (Cunliffe), *Gordialycus* sp., *Micropsammus* sp. 2, *Osperalycus tenerphagus* Bolton & Klompen; (ii) Leica DM2500 (DIC): *Asca* sp., *Leptus* sp., *Mexecheles hawaiiensis* (Baker); (iii) Leica Z16 APO (dissection microscope): *Hogna lenta* (Hentz); (iv) Olympus Spectral FV1000 (CLSM): *Micropsammus* sp. 1, *Nanorchestes* sp. 2, *Tydeus* sp.; (v) Phenom XL G2 Desktop (SEM): *Macrocheles muscaedomesticae* (Scopoli), *Oehserchestes* sp., *Proteonematalycus wagneri* Kethley; *Caenonychus* sp.; (vi) Zeiss Imager M2 (DIC): *Alycus* sp.; (vii) Zeiss LSM 710 (CLSM): *Nanorchestes* sp. 1, *Stigmalychus* sp.

Schematic representations (figures 1 and 7) were created using Blender (v. 3.0). The SEM procedures followed Bolton *et al*. [78] for the Hitachi S-4700 field emission SEM (cryo-SEM), and Bolton [79] for the Phenom XL G2 Desktop SEM. For DIC microscopy and CLSM, mites were mounted in either Hoyer's medium or polyvinyl alcohol. With respect to CLSM, excitation was via lasers of 405 nm (*Micropsammus* sp. 1 and *Nanorchestes* sp. 1) and 488 nm (*Nanorchestes* sp. 2, *Stigmalychus* sp. and *Tydeus* sp.). Autofluorescence was captured via broad detection bands from the wavelength of the laser up to around 650 nm. Solid, three-dimensional models were rendered in ImageJ [80] (v. 1.52 a), from which TIFF files of three-dimensional models were exported and labelled in Adobe Photoshop (v. 12.0). For *Tydeus*, a mesh model was also generated in order to obtain a good lateral view of the infracapitulum (figure 4c). The confocal *z*-stack was imported into 3D Slicer [81] (v. 5.4.0), where the threshold tool was used to automate the segmentation of the three-dimensional model in a single step. The model was then rendered in Blender (v. 3.6.1).

The complete dataset from the most taxonomically comprehensive (371 taxa) phylogenetic analysis of Acariformes to date [38] was analysed using identical methods, including the same partitions (rRNA: 18S and 28S; amino acids: COI, HSP70 and SRP54), models and software (IQ-TREE [82]), but with the single amendment of constraining Acari to be monophyletic (electronic supplementary material, treefile S4). A simplified version of the resulting tree was used for phylogenetic tracing of the state of the palpal coxae in Mesquite [83] (v. 3.8.1) using a parsimonious ancestral state reconstruction. The palpal coxae were coded as fused or unfused. Separation of the palpal coxae (a type of unfused state) was not treated as the alternative state to fused because the state of togetherness, meeting at the midline, does not necessitate fusion. Many non-mite arachnids (e.g. Solifugae, Opiliones and Pseudoscorpiones) have palpal coxae that meet at the midline but are unfused so that they can still freely articulate [18,30]. Coding was based on the taxonomic literature [32,34,36,41,45–50] and observations using mostly advanced techniques of microscopy (figures 2–4). Species in the phylogenetic analysis are not always conspecific with the species used for coding so that the state of the palpal coxae is treated as invariable throughout each genus. Phylogenetic tracing was also undertaken on simplified versions of trees from published analyses [37,38,84] (electronic supplementary material, figure S6). The procedure for these trees was the same as for the tree from the constrained analysis.

## Data Availability

Solid three-dimensional models that were generated with CLSM (Zeiss LSM 710 or an Olympus Spectral FV1000) are available in Dryad [85]. Supplementary material is available online [86].
